# Quality Assessment of Cancer Pain Clinical Practice Guidelines

**DOI:** 10.3389/fonc.2022.890951

**Published:** 2022-05-27

**Authors:** Zhigang Zhang, Xiao Cao, Qi Wang, Qiuyu Yang, Mingyao Sun, Long Ge, Jinhui Tian

**Affiliations:** ^1^ Evidence-Based Medicine Center, School of Basic Medical Sciences, Lanzhou University, Lanzhou, China; ^2^ Department of Intensive Care Unit, The First Hospital of Lanzhou University, Lanzhou, China; ^3^ Evidence-Based Nursing Center, School of Nursing, Lanzhou University, Lanzhou, China; ^4^ Evidence-Based Social Science Research Center, School of Public Health, Lanzhou University, Lanzhou, China; ^5^ The Key Laboratory of Evidence-Based Medicine and Clinical Transformation in Gansu Province, Lanzhou University, Lanzhou, China

**Keywords:** cancer pain, clinical practice guidelines (CPGs), quality, Reporting Items for Practice Guidelines in Healthcare checklist (RIGHT checklist), Appraisal of Guidelines for Research and Evaluation Instrument II (AGREE II)

## Abstract

**Introduction:**

Several clinical practice guidelines (CPGs) for cancer pain have been published; however, the quality of these guidelines has not been evaluated so far. The purpose of this study was to evaluate the quality of CPGs for cancer pain and identify gaps limiting knowledge.

**Methods:**

We systematically searched seven databases and 12 websites from their inception to July 20, 2021, to include CPGs related to cancer pain. We used the validated Appraisal of Guidelines for Research and Evaluation Instrument II (AGREE II) and Reporting Items for Practice Guidelines in Healthcare (RIGHT) checklist to assess the methodology and reporting quality of eligible CPGs. The overall agreement among reviewers with the intraclass correlation coefficient (ICC) was calculated. The development methods of CPGs, strength of recommendations, and levels of evidence were determined.

**Results:**

Eighteen CPGs published from 1996 to 2021 were included. The overall consistency of the reviewers in each domain was acceptable (ICC from 0.76 to 0.95). According to the AGREE II assessment, only four CPGs were determined to be recommended without modifications. For reporting quality, the average reporting rates for all seven domains of CPGs was 57.46%, with the highest domain in domain 3 (evidence, 68.89%) and the lowest domain in domain 5 (review and quality assurance, 33.3%).

**Conclusion:**

The methodological quality of cancer pain CPGs fluctuated widely, and the complete reporting rate in some areas is very low. Researchers need to make greater efforts to provide high-quality guidelines in this field to clinical decision-making.

## 1 Introduction

The global burden of cancer has risen to 19.3 million new cases and 10.0 million deaths in 2020 (1). Pain is one of the most common symptoms in over 70% of cancer patients, but nearly 50% of these patients are not well controlled, which has a negative impact on patients’ functional status and quality of life (2, 3). Therefore, it is necessary to adequately control pain and improve the condition of cancer patients under the guidance of evidence-based guidelines.

Clinical practice guidelines (CPGs) are statements that included recommendations aimed at improving patient care based on a systematic review of evidence and an assessment of the benefits and harms of alternative care options (4). Policymakers and clinicians generally regard CPGs as an essential tool for selecting the most evidenced and cost-effective treatments for practice. The purpose of the guidelines is to improve the quality of patient care by encouraging interventions with proven benefit and discouraging the use of ineffective or potentially harmful interventions; to reduce unnecessary variation in practice; to lessen disparities; and to influence public policy (5). A reliable guideline should follow strict methodological standards and provide complete and clear reporting (6, 7). Inadequately reported research may affect their reliability and application and may cause misunderstandings to target users (8). The Appraisal of Guidelines for Research and Evaluation (AGREE) II tool, as an international guideline evaluation gold standard, is the most widely used now (9). The Reporting Items for Practice Guidelines in Healthcare (RIGHT) checklist also has been widely implemented as a standard for guideline reporting (10).

At present, many international organizations have issued guidelines on cancer pain, aiming at providing guidance for alleviating the pain of cancer patients. Among these, the World Health Organization (WHO) analgesic ladder is widely used (11). Moreover, NCCN updates the *Adult Cancer Pain* every year (12). Although multiple cancer pain guidelines have been issued, so far, there have been no studies to critically evaluate the quality of existing cancer pain guidelines. Therefore, we used the AGREE II and RIGHT checklist to evaluate the methodology and reporting quality in order to identify high-quality cancer pain CPGs and explore potential strategies for improvement.

## 2 Methods

### 2.1 Study Design

We conducted a comprehensive review of clinical guidelines using the AGREE II instrument and RIGHT statement. This study was conducted and reported based on Preferred Reporting Items for Systematic reviews and Meta-Analyses (PRISMA).

### 2.2 Literature Search

We systematically searched Medline (*via* PubMed), Embase, Web of Science (WOS), Chinese National Knowledge Infrastructure (CNKI), WANFANG Database, Chinese Biomedical Literature Database (CBM), and VIP database to identify CPGs for cancer pain from their inception to July 20, 2021. We also searched the websites of the National Institute for Health and Care Excellence (NICE, https://www.nice.org.uk/), Guidelines International Network (GIN, https://guidelines.ebmportal.com/), National Guideline Clearinghouse (NGC, https://www.thecommunityguide.org/resources/national-guideline-clearinghouse), American Geriatrics Society (AGS, https://www.americangeriatrics.org/), Scottish Intercollegiate Guidelines Network (SIGN, https://www.sign.ac.uk/our-guidelines/), British Geriatrics Society (BGS, https://www.bgs.org.uk/), National Comprehensive Cancer Network (NCCN, https://www.nccn.org/), and World Health Organization guidelines (WHO, https://www.who.int/publications/i), as well as Google Scholar, MedSci (https://www.medsci.cn/), Dangdang (http://www.dangdang.com/), and Medlive (http://www.medlive.cn/) as supplemental sources. The search strategy combined the following terms: “cancer pain,” “cancer-related pain,” “practice guideline,” “guideline,” and “recommendation.” The details of search strategy are provided in **Appendix 1**.

### 2.3 Eligibility Criteria

We included CPGs that met the following criteria: (1) focused on the assessment, diagnosis, management, or treatment of cancer pain; (2) the CPGs were accessible or available; (3) published in Chinese or English; (4) the CPGs were the latest version; and (5) there is no restriction on the type of cancer pain. The following types of CPGs were excluded: (1) translations, interpretations, summaries, protocols, and the draft of guidelines; (2) older guidelines if a new version was accessible; (3) recommendations not only for cancer pain; and (4) expert consensus statements.

### 2.4 Selection and Data Extraction

Two pairs of reviewers (QW and MS, XC and QY) independently screened the titles and abstracts of the identified records. Then, the full texts of potentially relevant articles were downloaded and their eligibility further reviewed. Any discrepancies were resolved by discussion or through adjudication by a third reviewer (LG or JT).

Data of interest were extracted independently by four authors (QW, MS, XC, and QY) using a standardized electronic form. Data extraction included the first author, country, publication year, sponsoring organization, version (original or updated), grading system, development methodology (evidence-based or not), and funding. Disagreements were solved by consultation with another reviewer (ZZ). We conducted two rounds of pre-extraction to improve the consistency of the results.

### 2.5 Quality Appraisal of Guidelines

The methodological quality of the eligible CPGs was assessed by the AGREE II instrument (9). It consists of 23 items divided into six domains: “scope and purpose,” “stakeholder involvement,” “rigor of development,” “clarity of presentation,” “applicability,” and “editorial independence.” The score for each item ranges from 1 (strongly disagree) to 7 (strongly agree).

The reporting quality of the eligible CPGs was assessed by the RIGHT checklist (10). It contains 35 items divided into seven domains: “Basic information,” “Background,” “Evidence,” “Recommendations,” “Review and quality assurance,” “Funding and declaration and management of interests,” and “Other information.” The item was evaluated by “reported” (if the required information has been fully reported), “not reported” (if some required information was missing), or “not applicable” (if this item was not appropriate for assessing the specific CPGs).

Four reviewers (QW, MS, XC, and QY) independently assessed the quality of CPGs, and differences between the reviewers were resolved by consensus or consulting another reviewer (LG, JT, ZZ). Before a formal assessment, we conducted two rounds of pre-evaluation and discussed the appraisal criteria to ensure the consistency of results. Among the reviewers, MS and ZZ majored in nursing care, QW, XC, and QY participated in guideline development, and LG and JT are methodologists in guideline development. Therefore, most of the reviewers had rich experiences in guideline development or guideline evaluation, and some of them had experience of cancer patient care.

### 2.6 Statistical Analysis

Descriptive analysis was performed by calculating the value of each domain, total score, and standardized score by each reviewer based on AGREE II. The calculation formula of the domain score is as follows: (obtained score – minimal possible score)/(maximal possible score – minimal possible score) × 100%. The overall quality assessment score was based on the average score in all domains. The overall quality assessment of a guideline was appraised by four evaluators with a Likert scale from 1 to 7. The evaluator could give 6–7 points (if the score was more than 60% on all domains), 4–5 points (if the score was more than 30% on more than three domains but less than 60% on some domains), or 1–3 points (if the score was less than 30% on more than three domains). The guideline was used in practice to judge based on scores in six domains, and the AGREE-II manual recommends using “yes” (6–7 points), “yes with modifications” (4–5 points), or “no” (1–3 points). In addition, agreement among the four reviewers was determined by ICC, which was calculated by SPSS version 24.0 (IBM, Armonk, NY). The value of ICC ranged from 0 to 1. An ICC less than 0.10 was considered unreliable, that between 0.11 and 0.40 as mildly reliable, that between 0.41 and 0.60 as fairly reliable, that between 0.61 and 0.80 as moderately reliable, and that over 0.80 as indeed reliable (13).

We used Excel 2016 (Microsoft) to calculate the reporting rates for each item, domain, and overall, separately. In the calculation of the domain and the overall score, the proportion of 35 items is equal, and the items evaluated as “not applicable” are still included.

## 3 Results

### 3.1 Search Results

**Figure 1:**
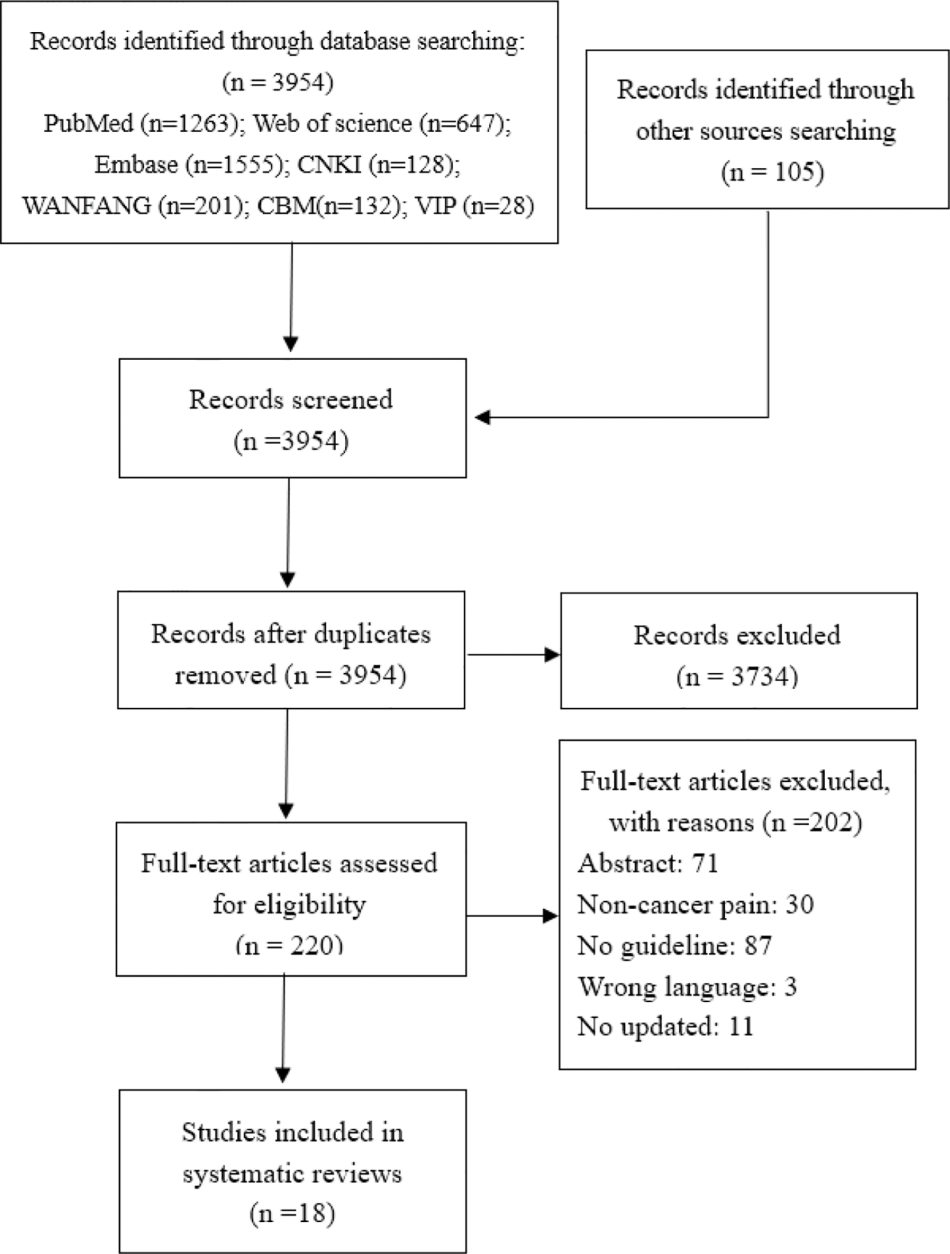
PRISMA flow diagram of study selection process.

Initially, we identified 3,954 records through database searching and 105 records through guideline websites. We excluded 3,734 studies after de-duplication and title screening and a further 202 after further screening the full text. Eighteen CPGs (14–31) proceeded to data extraction and synthesis. The selection process of the guideline is presented in **Figure 1**.

### 3.2 Guideline Characteristics

Of all the included CPGs, 11 CPGs (61.1%) (14, 15, 18–21, 24, 26, 27, 29, 31) were published in Europe, 4 CPGs (22.2%) (16, 23, 25, 30) were from the Americas, and 3 CPGs (16.7%) (17, 22, 28) were from Asia. Half of the CPGs (n = 10, 55.6%) were published after 2015. Eight CPGs (44.4%) (14, 15, 18, 22, 24–27) were updated versions, and the remaining 10 were original versions. There were two CPGs (11.1%) (15, 17) that did not conduct a systematical search, and seven CPGs (38.9%) (14, 16, 20, 25, 28, 30, 31) provided comprehensive search strategies. The recommendation of 13 CPGs (72.2%) (14, 16, 17, 19, 20, 22–25, 27–29, 31) focused on the management of cancer pain; recommendation of five other CPGs (27.8%) (15, 18, 21, 26, 30) focused on the treatment of cancer pain. Moreover, 13 CPGs (72.2%) reported the rating evidence criteria, and 12 CPGs (66.7%) provided the criteria for grading recommendations, in which the Grading of Recommendations Assessment, Development and Evaluation (GRADE) system is the most common method (five CPGs) (18, 20, 22, 26, 27). Among the 16 CPGs that have reported on the method of determining recommendations, the most common method is evidence combined with consensus meeting (six CPGs, 33.3%) (16, 18, 20, 27–29). **Table 1** summarizes the characteristic of the included CPGs.

**Table 1:** Characteristics of the cancer pain CPGs.

Sponsoring organization, year of publication	Country	Updated/original	Topic	Systematical search (yes/no)	Databases	Comprehensive search strategies(yes/no)	Criteria for rating evidence	Criteria for grading recommendation	Methods used to determine recommendations
ESMO, 2018 (14)	Europe	Updated	Management	Yes	MEDLINE (*via* PubMed)	Yes	Adapted from IDSA-USPHSGS	Adapted from IDSA-USPHSGS	Evidence + CC + modified Delphi
SEOM, 2017 (15)	Spain	Updated	Treatment	No	NR	No	Self-formulated (32)	Self-formulated (32)	NR
ASCO, 2016 (16)	USA	Original	Management	Yes	PubMed	Yes	NR	NR	Evidence + CC
CMH, 2013 (17)	China	Original	Management	No	NR	No	NR	NR	NR
EAPC, 2012 (18)	Europe	Updated	Treatment	Yes	CDSR, Medline, CENTRAL, EMBASE, CINAHL	No	GRADE	GRADE	Evidence + CC
EONS, 2014 (19)	Europe	Original	Management	Yes	NR	No	NR	NR	Evidence-based only
PCCWG, 2020 (20)	Netherlands	Original	Management	Yes	PubMed/MEDLINE, CINAHL, PsycINFO, HaPI, EMBASE, AMED, CENTRAL	Yes	GRADE	GRADE	Evidence + CC
APM, 2000 (21)	UK	Original	Treatment	Yes	CANCERLIT, Embase	No	Based on other study (33)	NR	Evidence-based only
JSPM, 2013 (22)	Japan	Updated	Management	Yes	PubMed, Cochrane	No	GRADE	GRADE	Evidence + modified Delphi
ASA, 1996 (23)	USA	Original	Management	Yes	NR	No	NR	NR	Evidence + ED
EAU, 2014 (24)	Europe	Updated	Management	Yes	Embase/Medline, CENTRAL, Eur-Lex, PsychInfo	No	OCEBM	OCEBM	Evidence + ED
NCCN, 2021 (25)	USA	Updated	Management	Yes	PubMed	Yes	NCCN categories of evidence and consensus	NCCN	Evidence + ED
NICE, 2016 (26)	UK	Updated	Treatment	Yes	PubMed or OVID’s MEDLINE	No	GRADE	GRADE	Delphi + nominal group method + CC +evidence + ED
WHO, 2018 (27)	Switzerland	Updated	Management	Yes	PubMed, Embase, CENTRAL, CDSR	No	GRADE	GRADE	Evidence +CC
ISSP, 2020 (28)	Indian	Original	Management	Yes	PubMed, Medline, CDSR, Google, OVID Search engine	Yes	NR	NR	Evidence + CC
SFORL, 2014 (29)	France	Original	Management	Yes	NR	No	ANAES	ANAES	Evidence + CC
CNS, 2021 (30)	USA	Original	Treatment	Yes	PubMed, EMBASE, CENTRAL	Yes	Self-formulated (34)	Self-formulated (34)	Evidence-based only
NCEC, 2015 (31)	Ireland	Original	Management	Yes	CDSR, Medline, CENTRAL, CINAHL, PsycINFO	Yes	OCEBM	OCEBM	Evidence + ED

ESMO, European Society for Medical Oncology; SEOM, Spanish Society of Medical Oncology; ASCO, American Society of Clinical Oncology; CMH, Cancer Pain Management Expert Panel of the Chinese Ministry of Health; CMH, Cancer Pain Management Expert Panel of the Chinese Ministry of Health; EAPC, European Association for Palliative Care; EONS, European Oncology Nursing Society; PCCWG, Pain in Children with Cancer Working Group; APM, Science Committee of the Association for Palliative Medicine of Great Britain and Ireland; JSPM, Japanese Society of Palliative Medicine; ASA, American Society of Anesthesiologists; EAU, European Association of Urology; NCCN, National Comprehensive Cancer Network; NICE, National Institute for Health and Care Excellence; WHO, World Health Organization; ISSP, The Indian Society for Study of Pain; SFORL, the French Otorhinolaryngology Head and Neck Surgery Society; CNS, Congress of Neurological Surgeons (CNS) and the Section on Pain; NCEC, The National Clinical Effectiveness Committee; CDSR, The Cochrane Database of Systematic Reviews; NR, not reported; GRADE, Grading of Recommendations Assessment, Development and Evaluation; CENTRAL, the Cochrane Central Register of Controlled Trials; CC, conference consensus; ED, expert discussion; IDSA-USPHSGS, the Infectious Diseases Society of America-United States Public Health Service Grading System; OCEBM, Oxford Centre for Evidence-based Medicine Levels of Evidence; ANAES, ANAES national health accreditation and assessment agency.

### 3.3 Methodological Quality of the Included Cancer Pain CPGs

**Figure 2:**
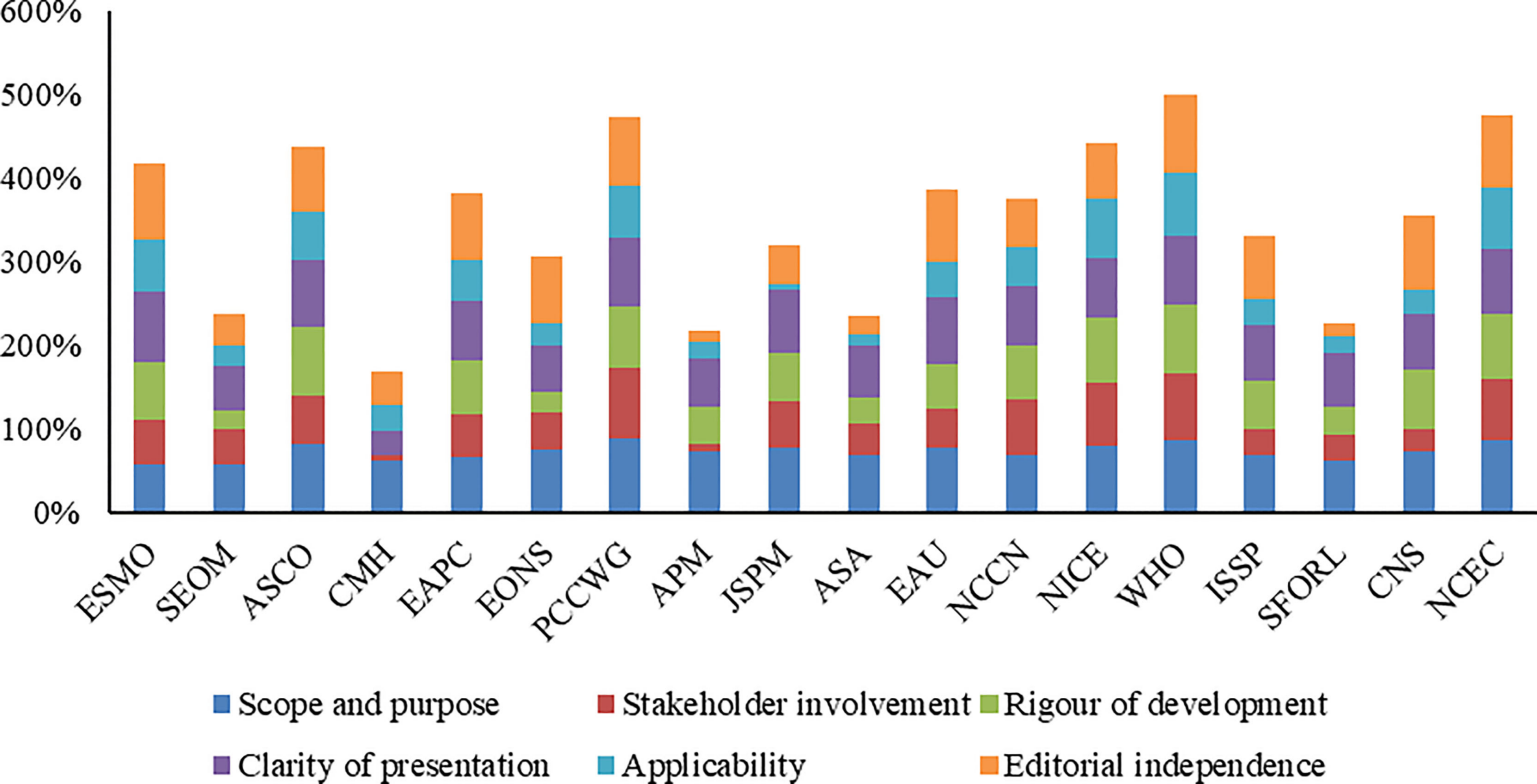
Total AGREE II score by domain across 18 guidelines.

The overall assessment and scores of CPGs assessed by the AGREE II tool are shown in **Table 2** and **Figure 2**. The ICC was substantial reliability for four domains (ICC >0.80) and moderate reliability for two domains (ICC = 0.76). Four CPGs (20, 26, 27, 31) were determined to be recommended, two CPGs (17, 21) not recommended, and the remaining 12 CPGs recommended with modifications. The highest mean score was 72% (range, 57% to 89%) on the “scope and purpose” domain, followed by 69% (range, 29% to 85%) on the “clarity and presentation” domain and 64% (range, 13% to 94%) on the “editorial independence” domain. The mean score on other three domains was less than 60%. The domains with low scores for all CPGs were “applicability,” “stakeholder involvement,” “rigor of development,” and “editorial independence.”

**Table 2:** Appraisal of Guidelines for Research and Evaluation (AGREE) II scores of cancer pain CPGs.

Sponsoring organization, year	Aspects of AGREE II evaluation (%)						Overall quality	Overall recommendation
	Scope and purpose	Stakeholder involvement	Rigor of development	Clarity and presentation	Applicability	Editorial independence	
ESMO, 2018 (14)	57	54	68	85	64	90	5	Yes, with modification
SEOM, 2017 (15)	57	42	23	54	24	38	4	Yes, with modification
ASCO, 2016 (16)	82	57	83	81	56	79	5	Yes, with modification
CMH, 2013 (17)	61	7	0	29	31	40	3	No
EAPC, 2012 (18)	65	51	65	72	47	81	5	Yes, with modification
EONS, 2014 (19)	75	44	24	57	25	81	4	Yes, with modification
PCCWG, 2020 (20)	89	83	73	82	63	83	6	Yes
APM, 2000 (21)	72	10	43	58	21	13	3	No
JSPM, 2013 (22)	78	54	58	76	6	48	4	Yes, with modification
ASA, 1996 (23)	69	38	30	63	15	21	4	Yes, with modification
EAU, 2014 (24)	78	47	53	79	42	88	5	Yes, with modification
NCCN, 2021 (25)	68	68	64	71	47	58	5	Yes, with modification
NICE, 2016 (26)	81	74	79	72	71	67	6	Yes
WHO, 2018 (27)	86	81	83	82	75	94	6	Yes
ISSP, 2020 (28)	68	31	59	67	30	75	4	Yes, with modification
SFORL, 2014 (29)	63	31	33	64	22	15	4	Yes, with modification
CNS, 2021 (30)	72	26	71	68	29	88	4	Yes, with modification
NCEC, 2015 (31)	86	74	78	78	73	88	6	Yes
Mean	72	48	55	69	41	64	–	–
ICC	0.76	0.82	0.94	0.76	0.84	0.95	–	–

ICC, intraclass correlation coefficient.

#### 3.3.1 Scope and Purpose

All CPGs scored over 50% for this domain. Most CPGs (16, 88.89%) clearly defined their objectives (item 1), health questions (item 2), and target populations (item 3). Only a limited number of CPGs (2, 11.11%) did not clearly define their health question and target populations.

#### 3.3.2 Stakeholder Involvement

Half of the CPGs (9, 50%) scored over 50% for this domain. Over half of the CPGs (10, 55.56%) clearly introduced their development group (item 4). Only a small number of CPGs (5, 27.78%) did not sought the views and preferences of the target population (item 5). Most CPGs (12, 66.67%) clearly defined the target users (item 6).

#### 3.3.3 Rigor of Development

Most CPGs (12, 66.67%) scored over 50% for this domain. Most CPGs (15, 83.33%) used systematic methods (item 7). Less than half of CPGs (8, 44.44%) clearly demonstrated the criteria for selecting the evidence (item 8). Most CPGs (13, 72.22%) clearly demonstrated the strengths and limitations of the body of evidence (item 9). More than half of CPGs (11, 61.11%) clearly described the methods for formulating the recommendations (item 10). More than half of CPGs (12, 66.67%) considered other factors in formulating the recommendations (item 11). Most CPGs (15, 83.33%) indicated a definite link between the recommendations and the supporting evidence (item 12). Less than half of CPGs (8, 44.44%) conducted an external review prior to release (item 13). More than half of CPGs (11, 61.11%) provided an updating procedure (item 14).

#### 3.3.4 Clarity of Presentation

Most CPGs (17, 94.44%) scored over 50% for this domain. Most CPGs (17, 94.44%) presented unambiguous and specific recommendations (item 15) and different options for managing the condition or health issue (item 16). Most CPGs (16, 88.89%) clearly identified key recommendations (item 17).

#### 3.3.5 Applicability

Only a limited of CPGs (6, 33.33%) scored over 50% for this domain. Only a limited of CPGs (6, 33.33%) described facilitators and barriers in application (item 18). Half of the CPGs (9, 50%) provided tools and/or advice to help the recommendations be put into practice (item 19). Only a limited of CPGs (7, 38.89%) considered the resources that may be required to implement the recommendations (item 20). More than half of CPGs (11, 61.11%) provided auditing and/or monitoring criteria (item 21).

#### 3.3.6 Editorial Independence

More than half of CPGs (12, 66.67%) scored over 50% for this domain. More than half of CPGs (12, 66.67%) declaimed that the funding agencies have not affected the content of the guideline (item 22). Most CPGs (15, 83.33%) collected and resolved competing interests of the guideline work group (item 23).

### 3.4 Reporting Quality of the Included Cancer Pain CPGs

**Figure 3:**
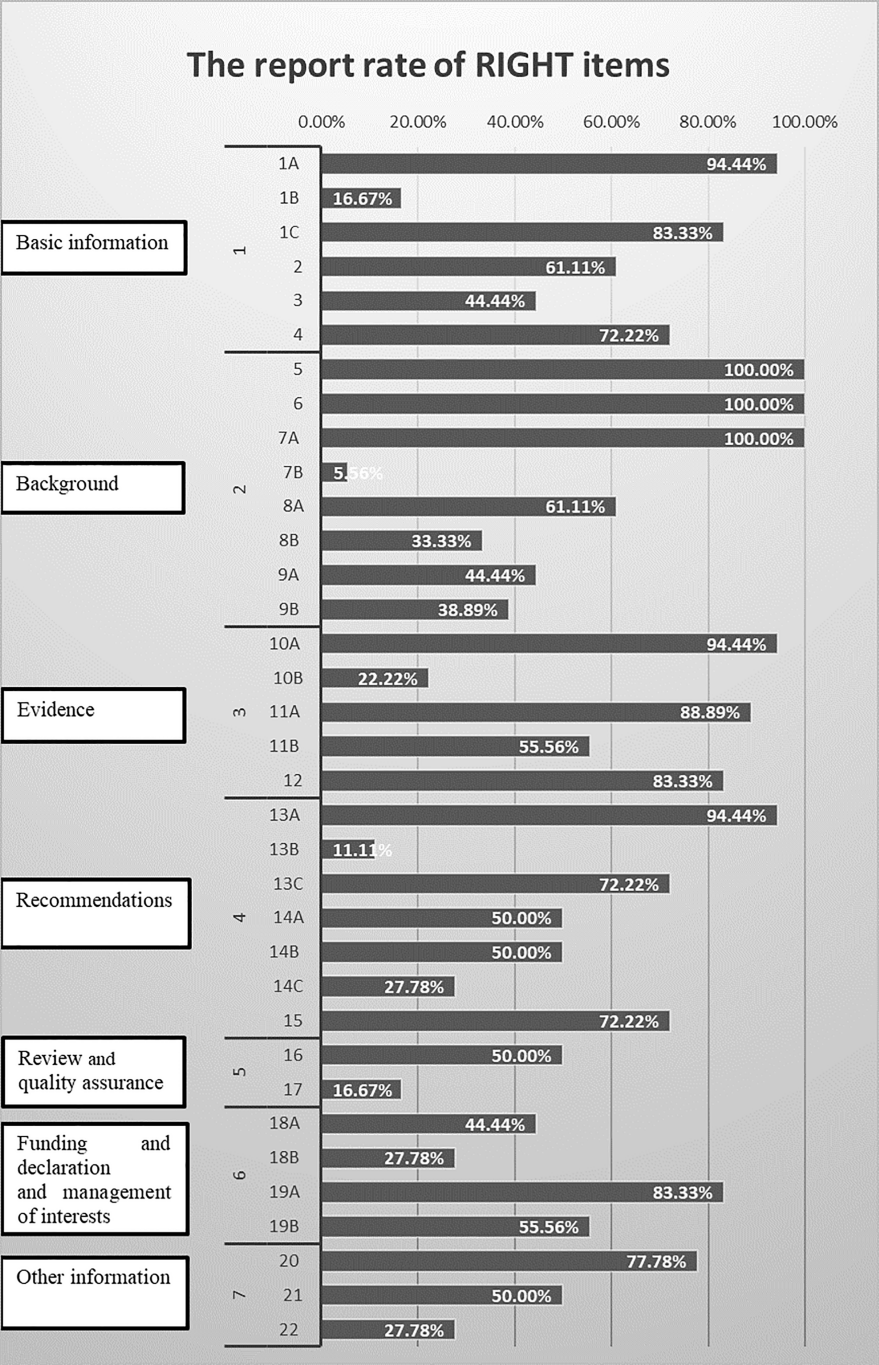
Reporting of RIGHT items in cancer pain guidelines.

**Table 3** presents the reporting rate of cancer pain CPGs according to the RIGHT statement. The average reporting rates of all CPGs was 57.46%. The highest rates domain was Evidence (domain 3) 68.89%, followed by Basic information (domain 1) 62.04%, Background (domain 2) 60.42%, Recommendations (domain 4) 53.97%, Funding and declaration of interest (domain 6) 52.78%, Other information (domain 7) 51.85%, and Review and quality assurance (domain 5) 33.3%. Only items 5, 6, and 7a were reported in all CPGs, which account for 8.57% of the 35 items. There were 62.86% items (22/35) reporting more than 50% of the CPGs. Nearly 6% of the CPGs reported item 7b (**Figure 3** **).**

**Table 3:** RIGHT domain score of the included cancer pain CPGs.

Sponsoring organization, year	Basic information (%)	Background (%)	Evidence (%)	Recommendations (%)	Review and quality assurance (%)	Funding and declaration and management of interests (%)	Other information (%)
ESMO, 2018 (14)	66.67	62.50	100.00	42.86	100.00	75.00	33.33
SEOM, 2017 (15)	66.67	37.50	60.00	57.14	0.00	25.00	33.33
ASCO, 2016 (16)	83.33	75.00	80.00	57.14	50.00	50.00	100.00
CMH, 2013 (17)	66.67	37.50	0.00	0.00	0.00	25.00	0.00
EAPC, 2012 (18)	50.00	50.00	80.00	57.14	0.00	75.00	100.00
EONS, 2014 (19)	33.33	50.00	40.00	14.29	0.00	75.00	0.00
PCCWG, 2020 (20)	83.33	62.50	100.00	85.71	0.00	100.00	100.00
APM, 2000 (21)	50.00	37.50	60.00	28.57	50.00	0.00	33.33
JSPM, 2013 (22)	66.67	75.00	60.00	42.86	50.00	25.00	66.67
ASA, 1996 (23)	66.67	62.50	40.00	28.57	0.00	0.00	33.33
EAU, 2014 (24)	66.67	75.00	60.00	85.71	0.00	100.00	33.33
NCCN, 2021 (25)	50.00	75.00	80.00	71.43	0.00	50.00	33.33
NICE, 2016 (26)	33.33	87.50	100.00	71.43	100.00	50.00	66.67
WHO, 2018 (27)	66.67	87.50	100.00	85.71	50.00	100.00	100.00
ISSP, 2020 (28)	66.67	37.50	80.00	57.14	50.00	50.00	33.33
SFORL, 2014 (29)	50.00	37.50	40.00	42.86	0.00	0.00	33.33
CNS, 2021 (30)	83.33	50.00	80.00	42.86	50.00	50.00	66.67
NCEC, 2015 (31)	66.67	87.50	80.00	100.00	100.00	100.00	66.67

#### 3.4.1 Basic Information

Overall, none of the included CPGs fully reported this domain. More than 50% CPGs reported the report type in the title (item1a, 17, 94%), the focus of the guideline (item 1c, 15, 83.33%), the summary of the recommendations (item 2, 11, 61.11%), and the corresponding developer or author (item 4, 13, 72.22%). Only three CPGs (item 1b, 16.67%) indicated the publication year, on the title, subtitle, cover page, or copyright page. Eight (item 3, 44.44%) CPGs provided a glossary or a list of acronyms or abbreviations. The descriptor of the report type included guideline(s) (n = 13), recommendation(s) (n = 2), management (n = 1), and various other terms (quick advice, manual, statement, toolkit, handbook, technical paper) (n = 1).

#### 3.4.2 Background

None of the included CPGs fully reported the background domain. One hundred percent of included CPGs reported the health problem (item 5), objectives (item 6), and target population (item 7a).

Eleven (61.11%) CPGs clearly reported the users of the guidelines (item 8a). Less than 50% CPGs reported subgroups (item 7b, 1, 5.56%), settings (item 8b, 6, 33.33%), and the roles and responsibilities of contributors (item 9a, 8, 44.44%) and individuals (item 9b, 7, 38.89%).

#### 3.4.3 Evidence

Only three (16.67%) CPGs reported all the items in the evidence domain. One of the included CPGs was not an evidence-based guideline, so it cannot evaluate most items of this domain. In 17 other CPGs, 17 (100%) CPGs stated the key question (item 10a), 16 (94.12%) CPGs were identified and assessed based on systematic reviews (item 11a), 10 (58.82%) CPGs indicated how those reviews (item 11b), 15 (88.24%) CPGs introduced the method for evaluating the certainty of evidence (item 12), and 4 (2.53%) CPGs explained how the outcome (item 10b) was selected and sorted.

#### 3.4.4 Recommendations

One of included CPGs was not an evidence-based guideline, so it cannot evaluate in this domain. Seventeen other CPGs (100%) introduced precise, clear, and operable recommendations (item 13a), 2 (11.76%) presented specific recommendations for important subgroups (item 13b), 13 (76.47%) reported both the strength of recommendations and the certainty of evidence (item 13c), 9 (52.94%) CPGs reported considering values and preferences (item 14a), 9 (52.94%) reported the cost and resource required to implement recommendation (item 14b), 5 (29.41%) described other factors (item 14c), and 13 (76.47%) reported the processes and methods used by the guideline work group for decision-making (item 15).

#### 3.4.5 Review and Quality Assurance

A total of 9 (50%) CPGs mentioned information on the external review of the final draft guidelines (item 16), and 3 (16.67%) CPGs reported the quality assurance program (item 17).

#### 3.4.6 Funding and Declaration and Management of Interests

The funding sources (item 18a) were reported in 8 (44.44%) CPGs, including 5 (27.78%) that described the role of funders in particular stages of development (item 18b). Interest declarations for all participants (item 19) were reportedly acquired in 15 (83.33%) CPGs, of which 10 (55.56%) reported the management of COIs (item 20).

#### 3.4.7 Other Information

A percentage of 77.78% of CPGs reported access to relevant documents (item 21), and 50% of CPGs reported discrepancies in evidence (item 22). Only 5 (27.78%) CPGs reported limitations (item 22).

### 3.5 Methodological and Reporting Quality by Publication Year and the Criteria for Rating Evidence

Overall, the AGREE II scores of the included CPGs showed a smooth trend in most domains according to publication year, which may be because most of the CPGs are evidence-based CPGs (**Supplement Figure S1**). Two CPGs which were published before 2000 had a low score in most domains, except the scope and purpose domain and the stakeholder involvement domain. The AGREE II score showed an obvious difference according to the criteria for rating evidence (**Supplement Figure S2**). The overall quality of CPGs using the GRADE approach was high. The overall quality of some guidelines that use other standards was moderate or even lower than those that do not use any standards. The RIGHT score showed an unclear trend according to publication year, which may be because the included CPGs are limited (**Supplement Figure S3**). The most significant increase was in the score of the “Evidence” domain, from 58.33% before 2018 to 90.00% after 2018. It experienced a slight improvement after the publication of RIGHT, but the overall quality of cancer pain is still moderate. The RIGHT score showed an obvious difference according to the criteria for rating evidence (**Supplement Figure S4**).The significant increase was in the score of the “Recommendations” domain, from 31.43% to 62.64%. Although cancer pain CPG has improved in most domains of methodology and reporting, the overall quality was still moderate.

## 4 Discussion

It is no doubt that improving the quality of the guidelines could play a large part in its advantages and promote the dissemination and implementation of the guideline. The guideline working group should follow the AGREE II to conduct a rigorous guideline development procedure and use RIGHT for detailed reporting. In recent years, the methodological quality and reporting quality of the cancer pain guidelines have been improved, but some aspects still need to be improved. We found that the methodological and reporting quality of cancer pain CPGs was highly heterogeneous among different domains even within the same guideline; the distribution of level of evidence and strength of recommendations also varied significantly among different categories of CPGs.

According to our findings on the methodological quality of the guidelines, unlike lung cancer CPGs, which score poorly in the domain of applicability and editorial independence, cancer pain CPGs do not score well in the applicability and stakeholder involvement domains (34). Compared with the methodological quality of nasopharyngeal carcinoma and oral cancer CPGs, cancer pain CPGs scored less than 50% not only in the applicability domain but also in the stakeholder involvement domain (35, 36). As with previous studies, this study also showed that all cancer-related CPGs scored low in the applicability domain, and there is a need to improve the quality of applicability and stakeholder involvement domains. According to the AGREE II result, two domains are in urgent need of improvement. Stakeholder involvement reflects whether CPGs better reflect the wishes of its target users, so it is necessary to include patient representatives in working groups or to conduct patient preference surveys. At present, the research on the patient preference survey is thriving, and it is feasible to increase patient preference in the guideline and recommend the optimal scheme according to patient preference. For the applicability domain, the guideline panels should conduct pre-surveys in advance to identify obstacles and facilitators in the implementation process and to provide appropriate responses and implementation criteria. Considering patient preferences and providing detailed implementation standards or manuals are more helpful for guidelines to be popularized in the field.

Based on our findings on the reporting quality of the guidelines, cancer pain CPGs only reported less than 50% in the review and quality assurance domain, which is different from the findings for lung cancer and gastric cancer CPGs with poor reporting rates for evidence, funding and declaration and management of interests, review and quality assurance, and other information (37, 38). The clinical practice guidelines on prostate cancer reported more than 50% only in the background and basic information domain (39). We think the good reporting quality of CPGs must be more than 50% of reporting rate. According to the RIGHT result, improvements are needed in three domains. Review and quality assurance was an important part of ensuring the high quality of CPGs. The possible reason may be that the readers and the authors may consider the rich recommendations to take precedence over the review process in CPGs. Limitations in the layout of journals can also lead to poor reporting. However, we believe that reports on improved review details and quality assurance methods will enhance the transparency of the review process, thereby improving the overall quality of the guideline working group; they need to report this part in its manuscripts or appendices. The recommendation domain, funding and interest declaration domain, and other information domain also needed more detailed reports. There is still a lack of reports related to explaining recommendations and describing the decision-making process; these items can help readers understand the recommendations easily and improve the recommendation quality. Therefore, dissemination and implementation of the guideline and recommendations in detail is necessary to include in the guideline. The report of funding and declaration of interest items can ensure the independence and impartiality of the CPGs. The report of other information items can improve the dissemination and implementation of CPGs. As we all know, the formulation of guidelines requires a lot of financial support; a reasonable and public financial support can ensure the fairness and credibility of the guidelines. Guidelines supported by national or provincial projects are bound to be more credible than those that rely on private funding. In addition, the low score of these cancer pain CPGs may be related to the lack of reports caused by periodical restrictions.

Currently published cancer pain guidelines focus on comprehensive management (e.g., the evaluation of cancer pain, basic principles, and adverse reaction management) or treatment only. The treatment guidelines focus on pharmacological interventions, with less mention of non-pharmacological intervention (such as psychological interventions, physical and complementary treatment). Nevertheless, based on our preliminary findings, with the improvement of cancer pain treatment measures, effective, convenient, and safe non-pharmacological therapies will become an increasingly important factor in future guidelines. For example, anxiety, depression, fear, and other psychological problems that come with cancer pain should not be underestimated. Several studies have reported that cancer patients pay attention to the psychological aspects associated with treatment (40–42). However, the analysis of the recommendations was not the purpose of this study. We expected that future studies could further analyze the recommendations of the guidelines based on multiple aspects (e.g., pharmacological, non-pharmacological treatments), in order to understand the recommendations of cancer pain in different areas and different interventions.

### 4.1 Strengths and Limitations

To the best of our knowledge, this is the first study to evaluate quality systematically for cancer pain guidelines. We strictly control the quality in the process of evaluation, carried out a comprehensive and systematic literature search, and obtained the highly unanimous opinion of the four evaluators which improved the credibility of our conclusion. Nonetheless, this study has some limitations. Due to the limitation of language skills, we only evaluated CPGs written in Chinese and English, which may affect the completeness of the current results. Moreover, evaluating the methodology and reporting quality of CPGs using the AGREE II and RIGHT checklists is a subjective process. Although we independently assessed and used ICC scores to detect consistency, bias was still possible. Meanwhile, due to limitations of the journal layout, the report of the guideline may indeed have some important information that leads to lower quality of the report. Furthermore, further analysis of the recommendations in these guidelines is also of interest. Despite these shortcomings, the consistency of comprehensive search and evaluation ensures the credibility of our research results.

## 5 Conclusion

This study found that more than half of the CPGs were of high quality that could be recommended. However, the reporting rate of existing cancer pain CPG is low in some domains, and its methodological quality is also inadequate. Researchers need to make greater efforts to provide high-quality guidelines for clinical decision-making.

## Author Contributions

ZZ: conceptualization, methodology, data curation, writing—original draft preparation; XC: conceptualization, methodology, data curation, writing—original draft preparation; QW: methodology, data curation, writing—original draft preparation; QY: data curation, writing—original draft preparation; MS: data curation, writing—original draft preparation; LG: writing—reviewing and editing, project administration; JT: supervision, project administration. All authors contributed to the article and approved the submitted version.

## Conflict of Interest

The authors declare that the research was conducted in the absence of any commercial or financial relationships that could be construed as a potential conflict of interest.

## Publisher’s Note

All claims expressed in this article are solely those of the authors and do not necessarily represent those of their affiliated organizations, or those of the publisher, the editors and the reviewers. Any product that may be evaluated in this article, or claim that may be made by its manufacturer, is not guaranteed or endorsed by the publisher.
