# Supplementary material for: Quality Assessment of Cancer Pain Clinical Practice Guidelines
Source: Front Oncol. 2022 May 27;12:890951. doi: 10.3389/fonc.2022.890951 (PMC9196585; doi:10.3389/fonc.2022.890951)
Supplement: Supplementary file 1 [file DataSheet_1.docx]

**Appendix A Search Strategy**

| **Databases [Platform]** *Searches run July 20, 2021* | **Results** |
| --- | --- |
| **PubMed** *July 20, 2021* | 1263 |
| **WOS** *July 20, 2021* | 647 |
| **Embase** *July 20, 2021* | 1555 |
| **CNKI** *July 20, 2021* | 128 |
| **CBM** *July 20, 2021* | 132 |
| **WanFang** *July 20, 2021* | 201 |
| **VIP** *July 20, 2021* | 28 |
| **Guideline sources** *July 20, 2021* | 105 |
| **Total** | 4059 |

**Pubmed**

| 1 | Cancer pain [MeSH Terms] | 1777 |
| --- | --- | --- |
| 2 | Cancer pain [Title/Abstract] OR cancer-related pain[Title/Abstract] | 8869 |
| 3 | 1 OR 2 | 9608 |
| 4 | "Practice guideline"[Publication Type] | 28835 |
| 5 | "Practice Guidelines as Topic"[MeSH Terms] | 124482 |
| 6 | "guideline"[Title/Abstract] OR "guidelines"[Title/Abstract] OR "recommendation"[Title/Abstract] OR "recommendations"[Title/Abstract] OR "statement"[Title] OR "guidance" [Title] | 650179 |
| 7 | 4 OR 5 OR 6 | 711671 |
| 8 | 3 AND 7 | 1263 |

**WOS**

| 1 | TS=(Cancer pain) OR TS=(cancer-related pain) | 52816 |
| --- | --- | --- |
| 2 | TI=(guideline) OR TI=(guidelines) OR TI=(recommendation) OR TI=(recommendations) OR TI=(statement) OR TI=(guidance) | 174365 |
| 3 | 1 AND 2 | 647 |

**EMBASE**

| 1 | 'cancer pain'/exp | 21590 |
| --- | --- | --- |
| 2 | 'cancer pain':ti,ab OR 'cancer-related pain':ti,ab | 14742 |
| 3 | 1 OR 2 | 27169 |
| 4 | 'practice guideline'/exp | 603097 |
| 5 | guideline:ti OR guidelines:ti OR recommendation:ti OR recommendations:ti OR statement:ti OR guidance:ti | 200845 |
| 6 | 4 OR 5 | 704390 |
| 7 | 3 AND 6 | 2072 |
| 8 | 'practice guideline'/de | 464003 |
| 9 | 7 AND 8 | 1555 |

**CNKI**

| # | Search Details | Results |
| --- | --- | --- |
| 1 | SU=指南 OR SU=指引 OR SU=草案 | 712543 |
| 2 | SU=癌性疼痛 OR SU=癌痛 OR SU=癌相关疼痛 OR SU=癌症相关疼痛 OR SU=瘤性疼痛 | 13581 |
| 3 | 1 AND 2 | 128 |

**CBM**

| # | Search Details | Results |
| --- | --- | --- |
| 1 | "指南"[不加权:扩展] OR "指南"[常用字段:智能] OR "指引"[常用字段:智能] OR "草案"[常用字段:智能] | 56051 |
| 2 | "癌性疼痛"[不加权:扩展] OR "癌性疼痛"[常用字段:智能] OR "癌痛"[常用字段:智能] OR "癌相关疼痛"[常用字段:智能] OR "癌症相关疼痛"[常用字段:智能] OR "瘤性疼痛"[常用字段:智能] | 19388 |
| 3 | 1 AND 2 | 132 |

**WANFANG**

| # | Search Details | Results |
| --- | --- | --- |
| 1 | 主题:指南 OR 主题:指引 OR 主题:草案 | 334515 |
| 2 | 主题:"癌性疼痛" OR 主题:"癌痛" OR 主题:"癌相关疼痛" OR 主题:"癌症相关疼痛" OR 主题:"瘤性疼痛" | 15620 |
| 3 | 1 AND 2 | 201 |

**VIP**

| # | Search Details | Results |
| --- | --- | --- |
| 1 | M=指南 OR M=指引 OR M=草案 | 107043 |
| 2 | M="癌性疼痛" OR M="癌痛" OR M="癌相关疼痛" OR M="癌症相关疼痛" OR M="瘤性疼痛" | 8506 |
| 3 | 1 AND 2 | 28 |

| # | **Other Sources** | Results |
| --- | --- | --- |
| 1 | Guidelines International Network (GIN) <https://guidelines.ebmportal.com/> | 6 |
| 2 | National Institute for Health and Care Excellence (NICE) <https://www.nice.org.uk> | 6 |
| 3 | National Guideline Clearinghouse (NGC)  <https://www.thecommunityguide.org/resources/national-guideline-clearinghouse> | 0 |
| 4 | Scottish Intercollegiate Guidelines Network(SIGN) https://www.sign.ac.uk/our-guidelines/ | 8 |
| 5 | British Geriatrics Society (BGS) <https://www.bgs.org.uk/> | 0 |
| 6 | American Geriatrics Society (AGS) <https://www.americangeriatrics.org/> | 0 |
| 7 | National Comprehensive Cancer Network (NCCN) https://www.nccn.org/ | 1 |
| 8 | World Health Organization guidelines (WHO) https://www.who.int/publications/i | 1 |
| 9 | Google | 41 |
| 10 | MedSci https://www.medsci.cn/ | 16 |
| 11 | 当当网“癌性疼痛指南” http://www.dangdang.com/ | 0 |
| 12 | 医脉通 “癌性疼痛”[过滤器：指南] <http://www.medlive.cn/> | 26 |

**Appendix B**

**Supplement Figure 1** AGREE II score according to the publication year

**Supplement Figure 2** AGREE II score according to the criteria for rating evidence

**Supplement Figure 3** Reporting rate of RIGHT items according to the publication of RIGHT

**Supplement Figure 4** Reporting rate of RIGHT items according to the criteria for rating evidence
